# Pharmacist-Led Interventions to Improve Medication Adherence in Older Adults: A Meta-Analysis

**DOI:** 10.1093/geroni/igab046.1295

**Published:** 2021-12-17

**Authors:** Zachary Marcum, Shangqing Jiang, Jennifer Bacci, Todd Ruppar

**Affiliations:** 1 University of Washington, Seattle, Washington, United States; 2 Rush University College of Nursing, Chicago, Illinois, United States

## Abstract

As pharmacists work to ensure reimbursement for chronic disease management services on the national (e.g., Medicare) level, summative evidence of their impact on important health metrics, such as medication adherence, is needed. The objective of this study was to assess the effectiveness of pharmacist-led interventions on medication adherence in older adults. In April 2020, a comprehensive search was conducted in six databases for publications of randomized clinical trials of pharmacist-led interventions to improve medication adherence in older adults. English-language studies with codable data on medication adherence and diverse adherence-promoting interventions targeting older adults (age 65+) were eligible. A standardized mean difference effect size (intervention vs. control) was calculated for the medication adherence outcome in each study. Study effect sizes were pooled using a random-effects meta-analysis model. Moderator analyses were then conducted to explore for differences in effect size due to intervention, sample, and study characteristics. The primary outcome was medication adherence using any method of measurement. This meta-analysis included 40 unique randomized trials of pharmacist-led interventions with data from 8,822 unique patients (mean age, range: 65 to 85 years). The mean effect size was 0.57 (95% Confidence Interval [CI]: 0.38-0.76). When two outlier studies were excluded from the analysis, the mean effect size decreased to 0.41 (95% CI: 0.27-0.54). Moderator analyses showed larger effect sizes for interventions containing medication education and when interventions had components delivered at least partly in patients’ homes. In conclusion, this meta-analysis found a significant improvement in medication adherence among older adults receiving pharmacist-led interventions.

